# Diverging responses to threats across generations in zooplankton

**DOI:** 10.1002/ecy.3145

**Published:** 2020-08-19

**Authors:** Yongcui Sha, Sylvie V. M. Tesson, Lars‐Anders Hansson

**Affiliations:** ^1^ Department of Biology Aquatic Ecology Lund University Lund SE‐22362 Sweden

**Keywords:** adaptation, *Daphnia magna*, multiple threats, predation, transgenerational plasticity, ultraviolet radiation

## Abstract

Our understanding on how organisms evolutionarily cope with simultaneously occurring, multiple threats over generations is still elusive. In a long‐term experimental study, we therefore exposed clones of a freshwater cladoceran, *Daphnia magna*, to threats from predation and ultraviolet radiation (UVR) during three consecutive parthenogenetic generations. We show that *Daphnia* can adapt to different sets of threats within three generations through modifying morphology, swimming behavior, or life‐history traits. When faced with predator cues, *D. magna* responded with reduced body size, whereas exposure to UVR induced behavioral tolerance when again exposed to this threat. Such UVR‐tolerant behavior was initially associated with a reduced clutch size, but *Daphnia* restored the reproductive output gradually through generations. The findings advance our understanding on how those common invertebrates, with a global distribution, are able to persist and rapidly become successful in a changing environment.

## Introduction

All animals on Earth are constantly faced with multiple and variable threats in their natural environments. In order to survive and reproduce, animals, as well as plants, have to handle the set of threats present in their local environment or respond behaviorally and move or migrate somewhere else. There are well‐known examples of such migrations, such as the mass‐migrations of ungulates on the African savannah and seasonal bird migrations in order to avoid low temperatures, shortage of food, or drought (Hansson and Åkesson 2014). In aquatic systems, large mass migrations of fish between lakes and rivers, likely driven by predation, have been noted (Brönmark et al. 2014). Moreover, millimeter‐sized zooplankton have long been known to perform diel vertical migrations (DVM) between surface and dark bottom waters to avoid predation and ultraviolet radiation (UVR; Hansson and Hylander 2009*a*, Williamson et al. 2011). However, if migration is not an option, an organism will have to acclimate to the local environment, for example, through phenotypic plasticity, or selection that eventually leads to genetic adaptation. Because any local environment exposes an organism to multiple threats, each of them varying temporally in strength, processes such as acclimation, plasticity, and selection will together mold the integrated phenotype (Pigliucci 2003), which can be seen as a compromise, or as an optimization, of the multivariate phenotype in a specific local environment. Although some aspects of the integrated phenotype may appear already within an individual’s lifetime as a plastic response, other coordinated trait responses may require exposure of multiple generations to emerge. To date, most studies on trait responses to local environments have focused on investigating single threats in isolation, and typically also quantified trait responses in a single generation of animals. Consequently, our understanding of transgenerational responses to multiple threats remains elusive. In order to fill this knowledge gap, we here aim at addressing the issue on how multiple threats affect the integrated phenotype over several generations; that is, both inter‐ (mother–offspring) and transgenerational (grandmother–mother–offspring) effects.

Predation is often among the most common and forceful of the multiple threats present in natural ecosystems. Besides the direct capture and ingestion by predators, resulting in an instant and complete loss of future fitness in the prey, perceived predation risk alone is powerful enough to affect the demographic structure of prey populations (Lima 1998, Creel and Christianson 2008, Zanette et al. 2011). For example, among songbirds fear of predation perceived by prey organisms results in a reduced reproduction rate (Zanette et al. 2011). In contrast to predation, UVR is not immediately lethal at low doses, but may cause detrimental effects on many organisms, both aquatic and terrestrial, owing to its highly energetic, short wavelengths (Rautio and Tartarotti 2010). For example, several studies show that exposure to UVR leads to reduced fecundity and higher mortality rates among zooplankton (Williamson et al. 1994, Rautio and Tartarotti 2010). However, in natural environments, common biotic and abiotic threats, such as predation and UVR, do not act in isolation but often occur simultaneously. This imposes considerable pressure on the organism, because a defense trait can be beneficial against one threat, but may increase the susceptibility to another. For example, when simultaneously exposed to predation and UVR, copepods reduced their pigmentation in response to predation but reducing photoprotective pigmentation made them more exposed to UVR (Hylander et al. 2009). To cope with these multiple threats, organisms have to evolve rapidly or respond plastically by modifying their behavior, morphology, and life‐history traits (Tollrian and Harvell 1999). Such plastic responses have been extensively studied in crustacean zooplankton, fish, and amphibians (Hansson and Hylander 2009*a*, Ferrari et al. 2010). However, most studies are built on short‐term experiments, rarely lasting longer than one generation of exposure to the threats, and may therefore fail to document the potential long‐term or transgenerational effects (Agrawal et al. 1999, Huebner et al. 2009).

Vertical migration is a common strategy among zooplankton to avoid predation and the UVR threat (Hansson and Hylander 2009*a*, Williamson et al. 2011). By spending the light hours in deeper and darker waters, they reduce the risk of being detected by visually hunting predators, such as fish (Lampert 1989, Hays 2003), as well as avoid high exposure to harmful radiation (Rhode et al. 2001). Zooplankton may also respond phenotypically, that is, protecting themselves from predation threats by changing their morphology, such as growing larger spines, neck‐teeth or helmets (Agrawal et al. 1999, Riessen and Gilbert 2019). The size‐efficiency hypothesis predicts that large individuals are stronger competitors because they are able to harvest more of the resources than small ones, whereas large individuals are generally a preferred prey for visually hunting predators, such as fish (Brooks and Dodson 1965, Hall et al. 1976). Hence, according to this hypothesis, large zooplankton may be removed by size‐selective predation from fish predators, leading to a zooplankton community dominated by small species (Brooks and Dodson 1965). Therefore, reducing body size may be adaptive for zooplankton in situations where large‐sized predators (e.g., fish) are dominant, which has been repeatedly shown for crustacean zooplankton (Brooks and Dodson 1965, Hall et al. 1976), but also for rotifers (Zhang et al. 2017*a*). Although such morphological acclimations driven by phenotypic plasticity may occur within a generation, it is unclear if the induction is further enhanced over generations, which seems to be the case with respect to handling toxic food by *Daphnia*. In an experiment where the herbivore *Daphnia* was exposed to cyanobacterial toxins, the granddaughters were less affected by the toxins than their mothers and grandmothers (Gustafsson et al. 2005); that is, the tolerance to the toxic food was enhanced through generations. However, our understanding of such adaptive transgenerational effects is scarce.

Cladocerans of the genus *Daphnia* have long been used as a model organisms for ecological and evolutionary research because of their wide distribution in nature. Members of this genus have succeeded in many water bodies with variable environmental threats, including predation and UVR. Notably, many *Daphnia* species are plastic within a single generation in their reaction to predator chemical cues (Boersma et al. 1998), as well as to harmful UVR (Rhode et al. 2001). However, there is now growing evidence demonstrating that phenotypic changes induced by environmental signals can span over multiple generations, a feature recognized as transgenerational plasticity (TGP) (Uller 2008, Jablonka and Raz 2009, Bonduriansky et al. 2012). Hence, any environmental threat experienced by parents may potentially alter the variation in their offspring’s phenotypes (Agrawal et al. 1999) and thereby provide them with an enhanced ability to tolerate future stressful conditions (Mousseau and Fox 1998). Such transgenerational responses have been documented in many organisms, including copepods, polychaetes and fish, which may respond to raising temperature, elevated levels of CO_2_, or hypoxia (Donelson et al. 2018). Yet few studies have investigated the multigenerational impacts of predation and UVR on crustacean zooplankton, despite their key role in aquatic ecosystems (but see Tanner and Branstrator 2006, Huebner et al. 2009, Santangelo et al. 2011), so the potential role of phenotypic plasticity, including transgenerational plasticity, in promoting *Daphnia* to adapt to these multiple threats over generations is still unclear.

Here we reared three clones of *Daphnia magna* under control (normal light), predation (normal light with fish cue), UVR, and the combination of predation and UVR, for three consecutive parthenogenetic generations. We then performed a series of assays that were designed to (1) measure the morphological responses of *D. magna* to single or multiple threats for each generation, (2) assess the swimming behavioral responses by *D. magna* from each treatment when again exposed to UVR threat, and (3) evaluate whether *D. magna* can become adapted to the new environment after three generations of acclimatization. By using asexually reproducing *Daphnia* as well as genetically identical individuals in different treatments to explore transgenerational responses, we could unambiguously attribute any variation among individuals within a clone line to plasticity. Therefore, we hypothesized that *D. magna* can distinguish between different threats and respond accordingly, suggesting that exposure to fish cue alone would lead to a reduced body size through generations. When exposed to a combination of fish cue and UVR, *D. magna* may change their body size or, alternatively, the combination with UVR may counteract this response. When exposed to UVR, we hypothesized that all individuals would exhibit avoidance behavior by swimming downward to deeper depths, and that individuals reared under UVR conditions (with or without fish cue) would show less response compared to naïve siblings (Hylander et al. 2014, Overholt et al. 2016). In addition, we recorded the number of offspring from the first two clutches for all individuals in each generation and used this as a proxy for reproductive output (physiological performance). This allowed us to test the hypothesis that the multigenerational exposure to UVR would enable *Daphnia* to adapt to the local environment of high stress and approach the original reproductive output eventually.

## Materials and Methods

### Clone collection and maintenance

This study included three clones of *D. magna*, which were collected with a plankton net (200 μm) from surface waters (0–0.5‐m depth) of a small fishless pond in Lund, Southern Sweden (55.6607 N, 13.5411 E). Each clone was established from a single parthenogenetic female. After isolation, three female *D. magna* adults were placed individually in separate 100‐mL glass jars filled with 60 mL copper‐free tap water at 20°C with a light regime of 30 μmol photons·m^−2^·s^−1^ and a 14 h light:10 h dark cycle in a controlled climate room. Because *Daphnia* are sensitive to copper, which may be released from copper pipes, we used copper‐free tap water delivered through plastic pipes throughout the study. Prior to the start of the experiment, the daphniids were maintained in laboratory for 4 months (at least five generations) and were fed every second day with 10^5^ cells of the microalga *Scenedesmus* sp. During these laboratory generations, animals were pipetted out once a week to allow for jar cleaning and the addition of new medium.

### Experimental design

We used a crossed design of fish predation and UVR which resulted in four treatments, including control (C: no UVR or fish cue), predation (P: no UVR but with fish cue), UVR (U: UVR without fish cue), and the combination of predation and UVR (PU: UVR with fish cue). The fish cue was prepared by rearing three crucian carps (*Carassius carassius*) in a 20‐L aquarium with aerated copper‐free tap water for 10 d and feeding them 100 *D. magna* every day. Water from this aquarium was filtered through a mesh size of 0.2 μm using vacuum filtration (10040‐436 VWR International, Radnor, Pennsylvania, USA) and immediately frozen to −20°C in 2‐mL microfuge tubes (20170‐170 VWR International). One microtube (2 mL) of this kairomone‐enriched medium was then melted and added to each of the replicates in treatments of P and PU every second day. For treatments of U and PU, *D. magna* were exposed to an intensity of UVR of 350 μW/cm^2^ during 14 h per day (photoperiod: 14 h light:10 h dark), which was provided by four UVA‐340 Q‐panel fluorescent lamps with a maximum emission in the UV‐A band (340 nm). A full spectrum description of the UV‐A panels is given in Hansson et al. (2007). In addition, four cool white tubes (Philips, 40 W, Eindhoven, The Netherlands) were also used to more closely mimic natural conditions (hereafter denoted “normal light”).

To initiate the experiment, we collected four female adults per clone and individually placed them into 100‐mL glass jars containing 60 mL copper‐free tap water. Four individuals of each clone were randomly assigned to the four treatments (C, P, U, and PU), constituting the initial generation (G0: 4 treatments × 3 clones × 1 replicate = 12 individuals). The mean size of individuals in the initial generation (G0) was 3.32 (± 0.09) mm. After they had reproduced, all the first asexual clutches were discarded and neonates (<12 h old) from the second clutch of each female were transferred individually into new jars with the same medium and food as their mothers in order to start the next generation (G1: 4 treatments × 3 clones × 3–11 replicates = 86 individuals). We repeated the same procedure for the next two asexual generations (G2: 4 treatments × 3 clones × 9–28 replicates = 179 individuals and G3: 4 treatments × 3 clones × 13–33 replicates = 288 individuals; Appendix S1: Fig. S1). Note that each of the three clones and generations were exposed to all the treatments. Therefore, we could control for the potential effect of genetic variation among experimental exposures, as well as across generations. A very low proportion of ephippial females was observed in the predation and UVR treatments throughout the experiment (Appendix S1: Fig. S2) but they also produced parthenogenetic clutches of female and male offspring. However, we only maintained females during the experiment and males were removed after identification under microscope. The experimental conditions were the same as described above (temperature: 20°C, photoperiod: 14 h light: 10 h dark). All individuals were fed with 10^5^ cells of the microalga *Scenedesmus* sp. every second day and were transferred to clean jars with new medium once a week.

The animals were checked for the presence of eggs every second day and the number of offspring was recorded for each individual when the first and second clutches were delivered. We used the sum of neonates produced during the first two clutches to estimate the reproductive output (hereafter denoted “clutch size”). We focused on the first two clutches because there were no age‐related changes in clutch size for any of the generations (Appendix S1: Fig. S3). Between 16 and 80 individuals from the three clones were monitored for each treatment and each generation, resulting in a total of 553 individuals (Appendix S1: Table S1).

### Tracking of swimming behavior

The *D. magna* females used for behavioral experiments were exposed to the respective treatment for 30–40 d after they were born. Between 6–38 adult females from each generation and each treatment with a total of 216 individuals were individually assayed for their behavioral response to the UVR (Appendix S1: Table S1). Prior to the trials, each individual was labeled using fluorescent nanoparticles (585 ITK Carboxyl Quantum dot, Life technologies, Prod. No. Q21311MP) following an adapted protocol from Ekvall et al. (2013). In short, one *D. magna* was exposed to 8 μL of the poly‐l‐lysine conjugated Quantum dot solution (Qdots) and then incubated in the dark at room temperature for 1 h. Labeled individuals were washed three times using filtered copper‐free tap water in order to remove excess Qdots. This unique method allows for tracking and behavioral assay of individual animals.

Behavioral tracking was performed in a Plexiglas aquarium (0.2 × 0.2 × 0.85 m), filled with 30 L of copper‐free tap water. Each labeled animal was individually introduced at the surface of the water in the aquarium using a 3 mL plastic Pasteur pipette and allowed to acclimatize for at least 10 min. However, occasionally some individuals swam down to the deeper depths or even bottom after the introduction. Therefore, we always waited to start the behavioral experiment until the animal was swimming in the upper 25 cm of the arena in order to standardize the initial UVR‐dose among all individuals. If the animal did not reach the upper 25 cm of the water column within 1 h the experiment with that specific individual was canceled. After acclimatization, we used four synchronized digital cameras (Pike F‐210C, Allied Vision Technologies GmbH) to record a 3‐min video for each individual. The video was divided into three phases: the first minute under blue excitation light only, the second minute with UVR turned on (150 mA, corresponding to a UVR intensity of 250 μW/cm^2^) and the last minute with UVR turned off. Three to five consecutive trials were performed with the same individual because of the technique constraints. Each individual was allowed to swim in the aquarium for 10–20 min before the start of the next trial. We used the mean of the first two complete tracks for later analyses. For more details about the tracking setup, see Palmér et al. (2016).

To avoid the cross‐contamination by the fish cue, a separate Plexiglas aquarium with the same size as described above was used to study the swimming behavior for individuals from the P and PU treatments. We set up the tracking aquarium 1 d prior to the behavioral assay by filling it with 27 L of copper‐free tap water and 3 L of fish cue from the aquarium with three crucian carp. Chemical cues released by fish evoke behavioral reactions in prey *Daphnia* (Hays 2003), although such reactions may only persist for a short period, for example, up to 6 h in the laboratory (Dodson 1988). Therefore, in order to ensure the presence of fish cue in the tracking arena, fresh fish cue was continuously diffused into the tracking aquarium during the video recording using a peristaltic pump (5 mL/min, ISMATEC®, Reglo ICC) which was connected to a side aquarium (10 × 10 × 20 cm) containing 1.45 L of copper‐free tap water and one crucian carp. Every morning, 30 *D. magna* were added as prey to this carp. At each tracking day, the behavioral experiment was performed from 9 a.m. to 4 p.m. and a total of 10–15 individuals were individually assayed per day. The tracking aquarium was emptied and replaced with new medium for the next tracking day.

We extracted the 3D position of each individual at six frames per second from their video recordings, according to the method described in Palmér et al. (2016) in MATLAB V1.7 and then calculated the swimming speed and the mean depth of each individual animal in the aquarium. When exposed to a UVR threat, the first reaction of an individual is generally to swim down, but it will stop at a certain depth where UVR is no longer dangerous. Therefore, we combined both the downward swimming and the chosen depth as refuge demand, which is calculated as the integral of an individual’s depth position over time (Fig. 2a; Hansson et al. 2016). We only focus on the refuge demand and swimming speed during the exposure to UVR, that is, during the second minute of each trial, to assess the behavioral differences in the UVR avoidance between individuals from each treatment and each generation. Hence, larger values of refuge demand are associated with individuals that behaviorally avoid UVR, whereas a small refuge demand indicates that the animals stay high up in the water column despite exposure to UVR (Hansson et al. 2016).

### Morphology

After the behavioral assay, each individual of *D. magna* was photographed with a camera (Infinity 1‐2CB) mounted on a microscope (Olympus SZX7). Two photos were taken for each *Daphnia*. Body length (from the highest point of the head to the base of the spine) was measured using the software ImageJ (version 1.52a, National Institutes of Health, Bethesda, Maryland, USA). The mean values of the measurements from those two photos were used for assessment of body length.

### Statistics

We only used one female adult per treatment and per clone to start the experiment and all the individuals from the generation 1 (G1) were exposed to different treatments before the formation of embryos. Therefore, all the initial mothers (G0) were excluded from the analyses because of the few replicates and also to remove the potential effect of treatment on the early growth. Variation in the body length, refuge demand, swimming speed, and clutch size was analyzed using linear mixed‐effect models (lme function in nlme package; Pinheiro et al. 2018). Treatment (four levels: C, P, U, and PU) and generation (three levels: G1, G2, and G3) were entered as fixed effects. Clone identification was entered as a random effect in order to control for unexplained variation among clones. Tukey’s post hoc pairwise comparisons were conducted to compare the levels of one factor within the levels of another, that is, how each response variable (morphology, behavior, and reproduction) changed over three generations within each treatment and for each generation how *D. magna* responded to different treatments (“lsmeans” package; Lenth 2016). Refuge demand and clutch size were transformed, respectively, using natural log function and square‐root transformation in order to meet the assumptions of the tests. For all models, a likelihood ratio test was used to determine the significance of the random effect. To investigate whether *D. magna* exhibited correlated morphological and behavioral responses during multi‐generational exposure to multiple threats of predation and UVR, we used Pearson’s correlation analyses between body size and behavior (refuge demand and swimming speed), combining all three generations. All the statistical analyses were performed in R v 3.5.0 (R Core Team 2018).

## Results

### Morphology

**Fig. 1:**
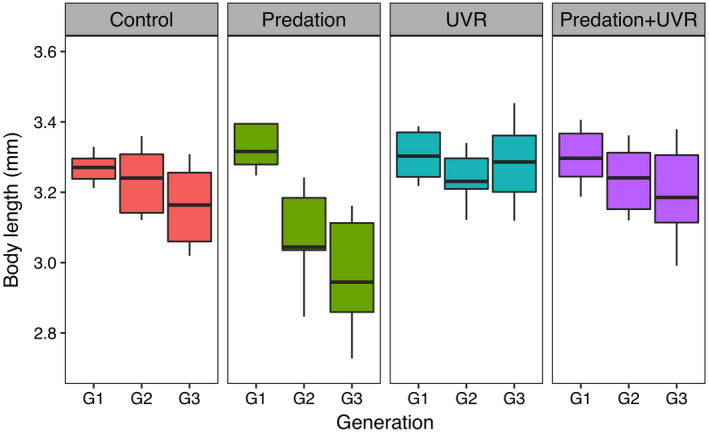
Transgenerational changes in body length of *Daphnia manga* under treatments of control, predation, UVR, and the combination of predation and UVR from generation 1 to 3 (G1–G3). Boxes show the first and third quantiles, lines within boxes show mean values, and whiskers show ± SD.

The transgenerational changes in body size were strongly associated with the treatment the animals were exposed to, manifested in a significant interaction between treatment and generation (Table 1). When exposed to fish cue, *D. magna* reduced their mean body size with more than 11% from generation 1 to 3, whereas there were no significant changes in body size through generations among the other treatments (C, U, and PU, Figs. 1 and 4a, Appendix S1: Tables S1 and S2). Clone random effect was significant (Table 1), suggesting that changes in body size varied significantly among clones of *D. magna*.

**Table 1:** Results of the linear mixed effects model analyses on morphology (body length), behavior (refuge demand and speed) and reproduction (clutch size) of *Daphnia magna*.

	df	Body length	Refuge demand	Speed	Clutch size
		*F* (df)	*F* (df)	*F* (df)	*F* (df)
Fixed effects					
Treatment	3	**23.924** * **(231)**	**16.286** * **(202)**	**6.223** * **(202)**	**11.286** * **(539)**
Generation	2	**12.201** * **(231)**	2.179^NS^ (202)	1.265^NS^ (202)	**24.900** * **(539)**
Treatment:generation	6	**3.967** * **(231)**	0.499^NS^ (202)	0.229^NS^ (202)	**3.444** * **(539)**
Random effects					
Clone	1	**6.333** *	**6.468** *	0.320^NS^	**11.923** *

Notes:

The denominator degrees of freedom are displayed after each *F* value. Bold font indicates significant results (*P* < 0.05). Number of replicates for each treatment and generation are reported in Appendix S1: Table S1.

* *P* < 0.05; **P < 0.01; ***P < 0.001; ^NS^ *P >* 0.05.

### Behavior

**Fig. 2:**
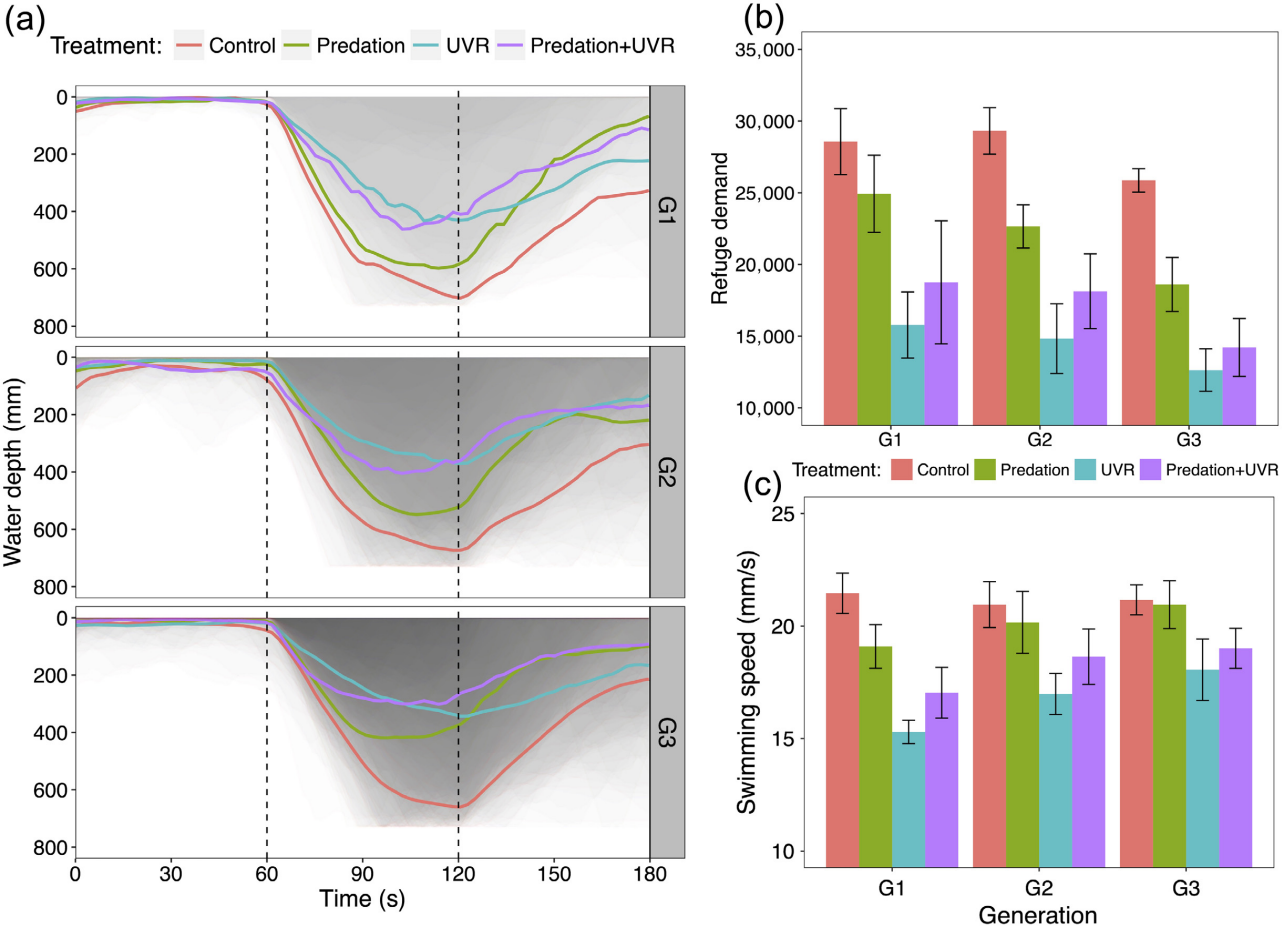
Behavioral responses of control, predation, UVR, and the combination of predation and UVR treated *Daphnia magna* from generation 1 to 3 (G1–G3). (a) Average (thick lines in different colors) and individual (overlapping gray areas) vertical position (mm) during three UVR phases: 1 min (0–60 s) acclimatization, then 60 s with UVR switched on, and then 60 s without UVR. (b) Refuge demand (mm × s) during UVR exposure (the gray areas of the middle 1 min in (a). (c) Mean differences in swimming speed when individuals were exposed to UVR. Individuals which previously had experienced UVR swam slower at the initial generation but the differences decreased at the last generation. Red, green, blue, and purple lines/areas represent control, predation, UVR, and the combination of predation and UVR treatments, respectively. Bars and whiskers show mean ± SE.

All individuals generally gathered close to the surface during the first minute (without UVR, resembling “night conditions”) and showed a rapid downward movement when the UVR was switched on (resembling day conditions; Fig. 2a). However, individuals raised under UVR (U and PU treatments) showed significantly less response than animals raised without UVR (C and P treatments; Fig. 2a, b). During the last minute, that is, the recovery phase from UVR, animals from all generations and different treatments showed a tendency to return to the surface (Fig. 2a). The refuge demand showed a nonsignificant decreasing trend through generations for all treatments (Table 1; Fig. 2b; Appendix S1: Table S3). However, irrespective of generation, animals raised under control and predation treatments showed similar refuge demands, which were significantly larger than for animals raised under UVR conditions, but no difference was found between U and PU (Figs. 2b, 4c; post hoc tests, C‐P: *P* = 0.397; C‐U: *P* < 0.001; C‐PU: *P* < 0.001; P‐U: *P* < 0.050; P‐PU: *P* < 0.050; U‐PU: *P* = 0.995). Moreover, UVR‐induced individuals always showed less of a response to UVR compared to control individuals (Figs. 2a, 4c, Appendix S1: Tables S1, S3).

We calculated the response in swimming speed to UVR for each individual, showing that generation had no effect on the speed for any treatment (Table 1); offspring swam with similar speed as their mothers. However, treatment induced different speed responses to UVR (Table 1; Fig. 2c), where the control animals showed the highest speed (Fig. 2c). The animals previously exposed to UVR showed the lowest speed response when again exposed to UVR throughout generations. However, differences in speed among treatments decreased through generations and all individuals showed almost similar speed responses after three generations (Figs. 2c, 4d; Appendix S1: Tables S1 and S4).

In addition, we also observed significant variation among clones for refuge demand, however, we did not find evidence for variance among clones for swimming speed (Table 1).

### Reproduction

**Fig. 3:**
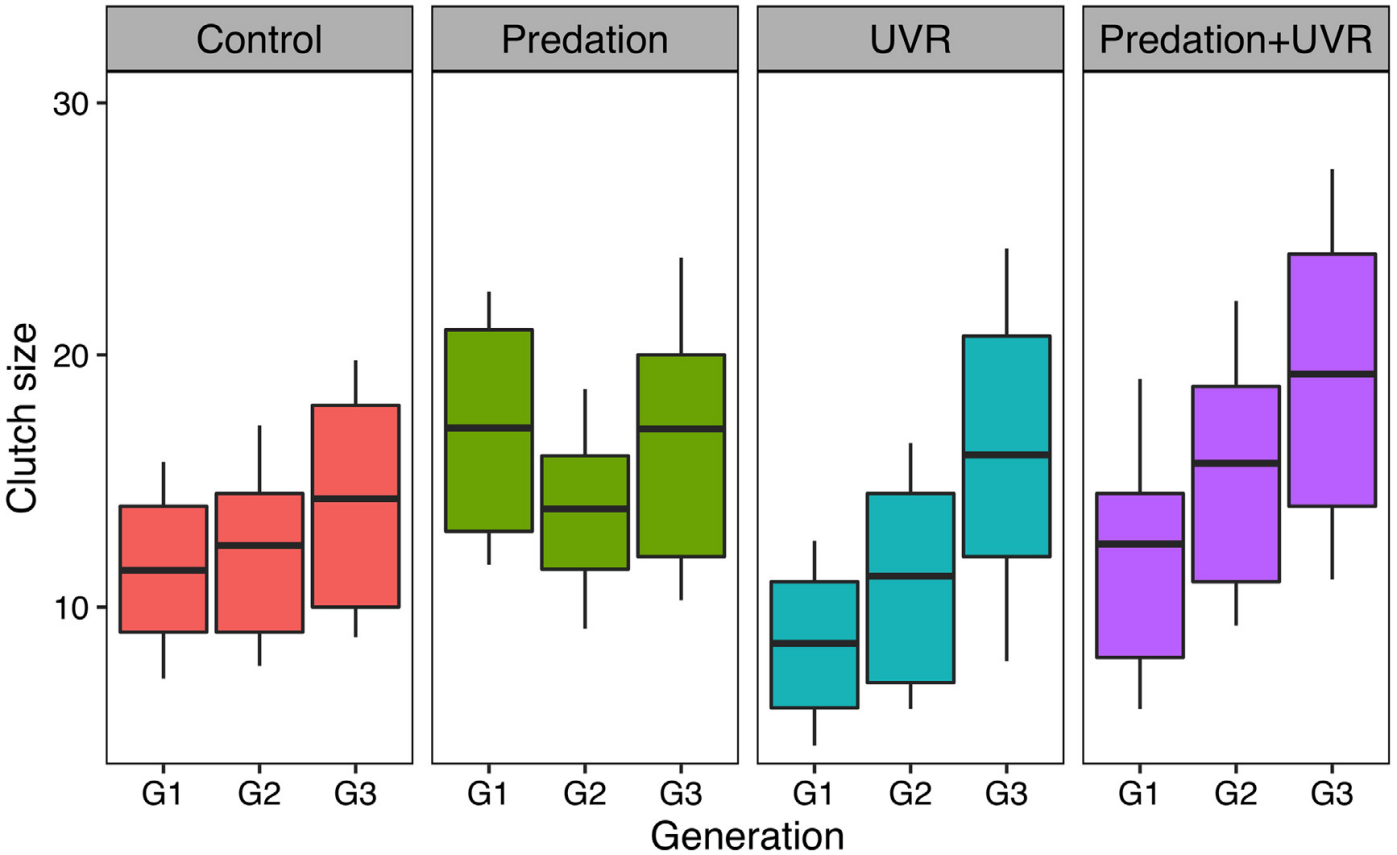
Transgenerational responses in clutch size of *Daphnia magna* exposed to the treatments: control, predation, UVR, and the combination of predation and UVR. Boxes show the first and third quantiles, lines within boxes show mean values, and whiskers ± SD.

**Fig. 4:**
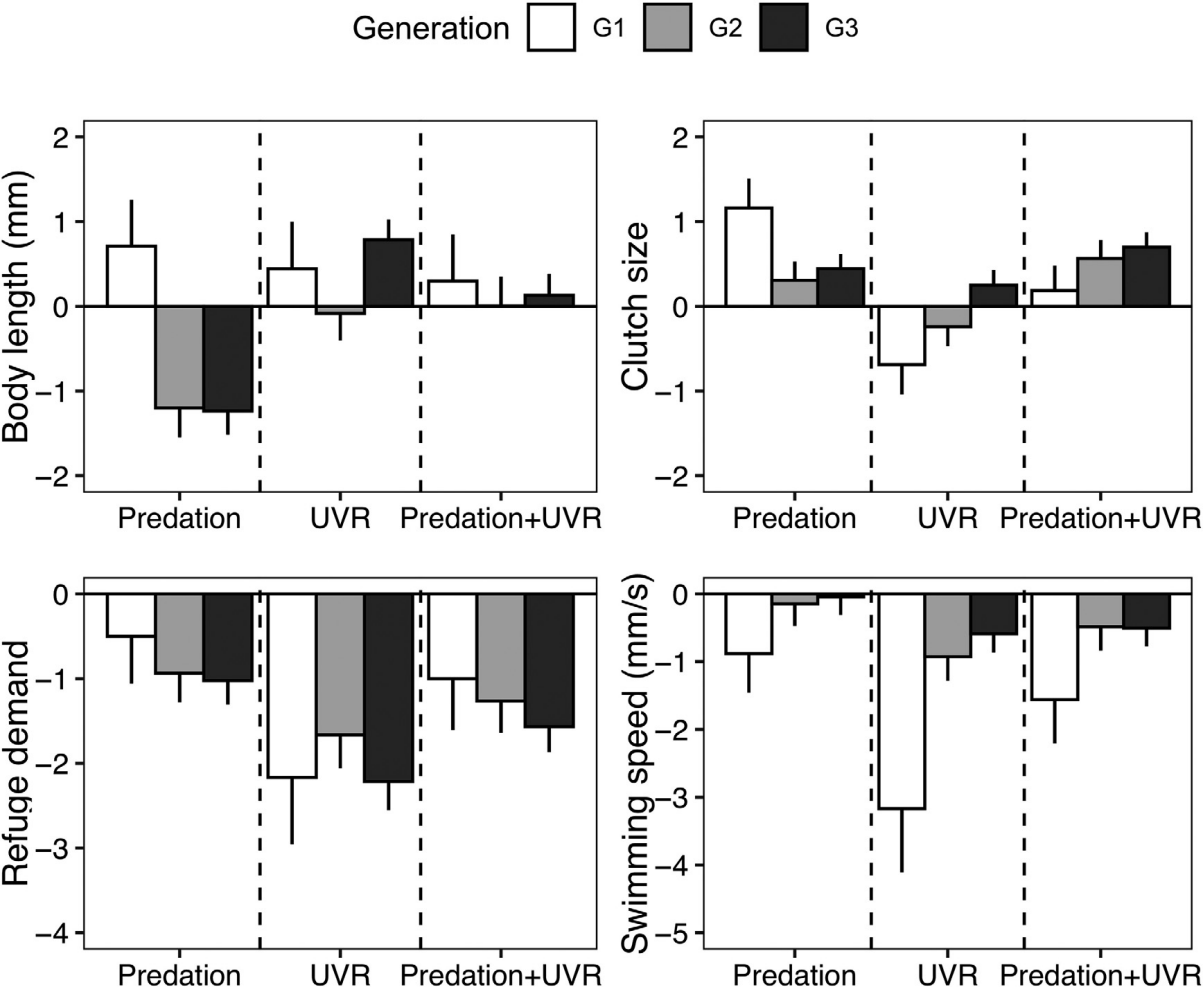
The mean effect sizes (Cohen’s *d*) of (a) body length, (b) clutch size, (c) refuge demand, and (d) speed for *Daphnia* individuals under treatments of predation, UVR, and the combination of predation and UVR. Bars show the development during three consecutive generations (G1, white bars; G2, light gray bars; G3, dark gray bars), which were normalized using the mean value of control individuals as zero baseline. Error bars are standard errors.

There was a strong interaction between treatment and generation regarding clutch size (Table 1). Under normal light without fish cue (control treatment), we found a nonsignificant increasing trend in the clutch sizes through generations (Appendix S1: Table S5). When exposed to predation treatment, *D. magna* consistently had high clutch sizes over three generations (Figs. 3 and 4b). However, animals exposed to UVR showed significantly smaller clutch sizes at the first generation (G1, post hoc tests, Appendix S1: Table S5), but the clutch sizes eventually increased through generations (Figs. 3 and 4b). Hence, at G3, *D. magna* from all treatments had similar clutch sizes of about 14–19 neonates per female (Fig. 3; Appendix S1: Table S1). We also found a significant effect of clone on clutch size (Table 1).

### Correlation analyses between morphology and behavior

**Fig. 5:**
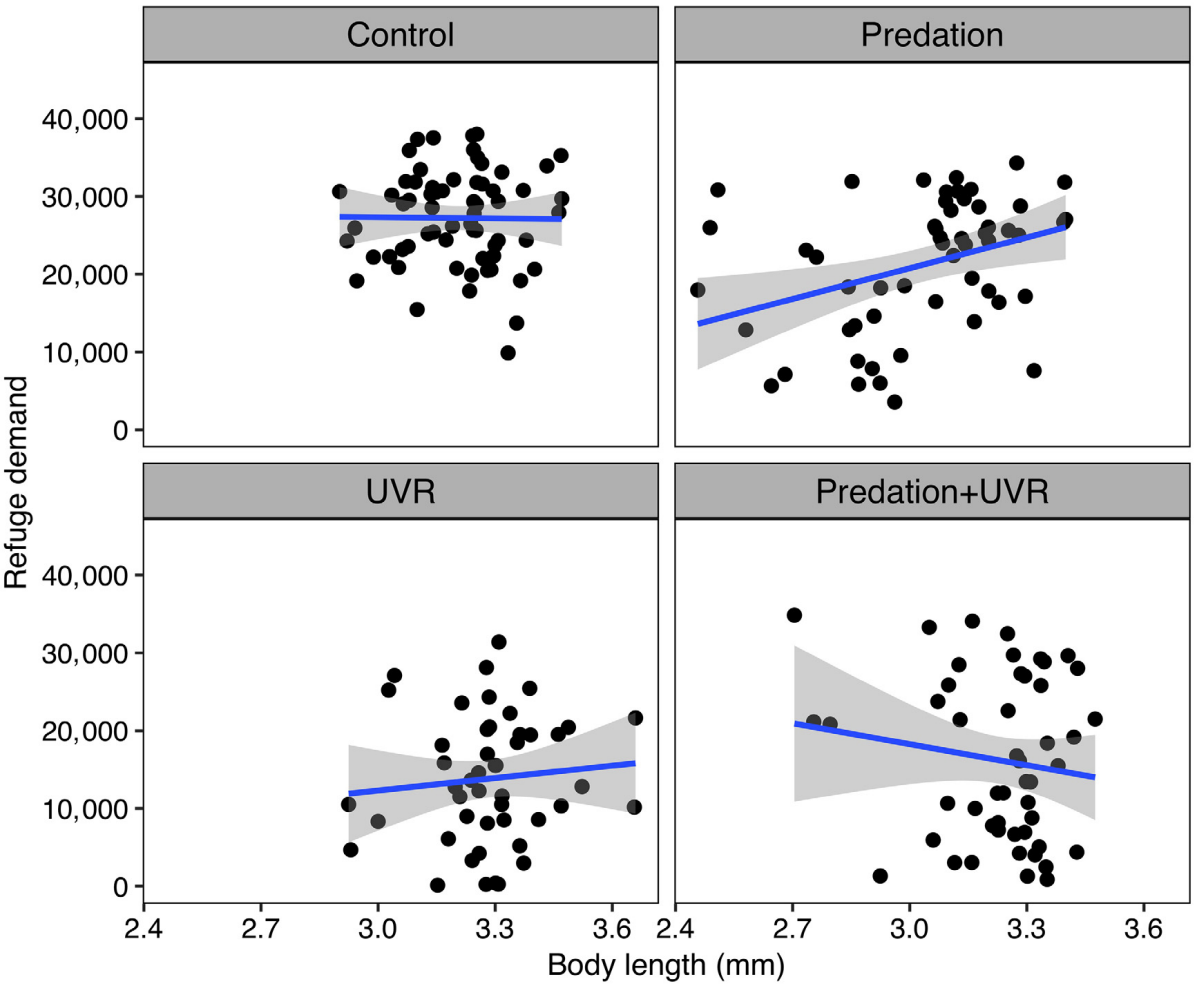
Relationships between refuge demand and body length for treatments of control, predation, UVR, and the combination of predation and UVR. The graphs also show the linear regression lines and the confidence intervals as gray shaded area.

In order to investigate the potential relation between behavior and morphology further, we performed correlation analyses between refuge demand and size of animals, as well as speed and size of animals throughout generations. A significant positive correlation was found between refuge demand and body length in the predation treatment (*r* ^2^ = 0.360, *P* < 0.010, Fig. 5); that is, larger individuals had a higher refuge demand than smaller when exposed to predation, but not so in any other treatment. On the contrary, there was no correlation between swimming speed and body length when fish cue was present (P: *r* ^2^ = 0.144, *P* = 0.298; PU: *r* ^2^ = 0.056, *P* = 0.703). When fish cue was absent (C and U treatments), there was a nonsignificant tendency for a positive relation between speed and body length (C: *r* ^2^ = 0.239, *P* = 0.055; U: *r* ^2^ = 0.267, *P* = 0.070).

## Discussion

By using a multigenerational exposure experiment, we investigated changes in the transgenerational response of *D. magna* to fish predation and UVR, both separately and in combination, and the results demonstrate that *D. magna* are able to detect and distinguish between different types of threats and respond accordingly by modifying their morphology, swimming behavior, and life‐history traits across generations. We also fill a knowledge gap by showing that, over generations, the responses in behavior and morphology to one of the threats are not the same as when predation and UVR are combined. Instead, a combination of threats leads to an integrated phenotype already after three generations.

In accordance with previous short‐term studies (Riessen 1999, Zhang et al. 2017*a*), we found a significant decline in body size of *D. magna* through generations when exposed to fish cue (P treatment). Specifically, *D. magna* from the third generation showed the smallest mean body size, which were 3 and 11% smaller than their mothers (G2) and grandmothers (G1), respectively, suggesting that the maximum morphological response to fish cue may require several generations of exposure as reported by Tanner and Branstrator (2006). This result is also consistent with the size‐efficiency hypothesis (Brooks and Dodson 1965). Given that planktivorous fish are size‐selective predators, that is, they preferentially forage on prey items of larger sizes, a smaller body size will allow daphniids to be less visible and less vulnerable to fish predation. Hence, in our study of *D. magna* individuals not exposed to predators for at least 100 generations, it may be assumed that the selection has favored large individual size, and thereby a phenotype optimal for competition. However, we show here that already after three generations of exposure to predator (fish) cues, the individual size had decreased by 11%. Hence, we show, for the first time, that not only selective predation on large individuals, which was the mechanism originally suggested for the size differences in the presence and absence, respectively, of predators (Brooks and Dodson 1965), but also that the presence of predator cues induces a decreasing size in the prey. Hence, our result adds another, nonexclusive explanation to the size‐efficiency hypothesis (Brooks and Dodson 1965), namely, that the prey actually induces a smaller body size in order to become less vulnerable to predation, a mechanism that has also been demonstrated for rotifers (Zhang et al. 2017*b*). In addition to a reduction in body size, individuals cultured in the presence of fish cues (normal light, P) also responded with increased clutch size compared to control individuals. Many animals, including killifish (Grégoir et al. 2018), some songbirds (Mönkkönen et al. 2009) and even mammals (e.g., a wild rodent, *Myodes glareolus*; Haapakoski et al. 2018), have been found to produce more offspring under the threat of predation. In our study, predatory cues induced trait shifts, including a smaller size and higher reproductive output, which may be associated with an energetic trade‐off between somatic growth and reproduction, where *Daphnia* allocate more energy into a higher production of offspring in the presence of fish that may enhance the fitness of the individual in a situation with high predation risk.

In contrast to the response to predation, UVR did not lead to any changes in body size, but instead altered the individual swimming behavior. When dwelling at the surface where the intensity of UVR was always very strong, the most common way of avoiding harmful radiation for zooplankton is to move away and swim deeper down, because UVR attenuates and diminishes with depth (Scully and Lean 1994). Thus, downward swimming would provide zooplankton with a refugia from UVR. Accordingly, in our study, all individuals showed a rapid downward swimming when exposed to UVR, but the strength in response differed between treatments where individuals reared under UVR conditions (with or without fish cue) had a smaller refuge demand and also swam slower than their naïve siblings (treatments of C and P). The control individuals displayed the strongest response with about a doubling in refuge demand compared to the UVR‐raised individuals, suggesting that individuals exposed to UVR acquired tolerance after one generation of exposure to the threat. Individuals reared under fish cue (P treatment) showed a similar response pattern as control individuals, suggesting that previous experience of exposure to UVR was the major force determining the depth distribution of *D. magna*. These results are consistent with previous studies, showing that zooplankton, including copepods and *Daphnia*, show a more relaxed behavioral response to UVR when they have prior experience of the threat (Hylander et al. 2014, Overholt et al. 2016). The potential explanations for the tolerant threat response may be due to the increased amounts of photoprotective pigmentation, such as melanin (Hansson et al. 2007) and also other defensive strategies such as enhanced photoenzymatic repair (Hansson and Hylander 2009*a*), which may protect the nonpigmented individuals avoiding UVR damage.

UVR exposure also changed the reproductive strategy as individuals reared under UVR stress (with or without fish cue) showed an increased clutch size through generations. The significantly reduced clutch size during the initial exposure period (generation G1 and G2) may be due to the strong UVR‐induced oxidative damages on proteins, lipids, and DNA (Oexle et al. 2016). Another reason may be that *D. magna* allocated energy from reproduction to photoenzymatic repair or some other mechanisms to compensate for UVR damage and therefore maintain growth, because adult size was not affected by exposure to UVR.

Context‐dependent trait variation, such as phenotypic plasticity, acts within a generation, but is generally assumed not to be transferred to the next generation. However, recently transgenerational plasticity (TGP), wherein the local environment may induce phenotypic changes persisting over several generations, has been suggested as a link between phenotypic plasticity and evolutionary processes (Walsh et al. 2016). TGP may buffer against the negative effects of environmental changes, as Rodríguez‐Romero et al. (2016) reported that a marine polychaete restored their fecundity to a normal level after three generations under low *p*CO_2_ conditions. This is also the case in our study, where we found that from G1 to G3 the clutch sizes increased by 69 and 54% in the U and PU treatments, respectively. Similarly, Fernández et al. (2018) also found that *Daphnia* populations exposed to high levels of UVR had higher fecundity and earlier reproduction than at control conditions. Therefore, increased clutch size may be an alternative strategy to handle high mortality caused by UVR, which enables the population to persist under harsh environmental conditions with high UVR exposure.

We found no change in body size through generations when *D. manga* were simultaneously exposed to predation and UVR (PU treatment). One possible reason is that UVR may affect the efficiency of fish cue and thereby reduce the predator‐induced responses (Sterr and Sommaruga 2008), or that *Daphnia* may not respond to predation but instead invest more energy to produce suitable protection strategies against UVR. A previous study has also found that UVR can affect the expression of antipredator morphology, as Alton et al. (2010) observed that tadpoles did not morphologically respond to predator cues when simultaneously exposed to UVR. Therefore, UVR may interact with predation and indirectly reduce the zooplankton survival under predation by suppressing the development of inducible morphological defenses.

Interestingly, when considering the body size of individuals reared without fish cue (treatment of C and U), there is a marginally significant tendency that large individuals swim faster in response to UVR compared to smaller ones (*P* < 0.100), but they were evenly distributed among depths no matter of which size class they belonged to. A similar result was also reported in Hylander et al. (2014) who found smaller daphniids to swim slower than larger ones. However, individuals reared in the presence of fish cue (P treatment) showed a different response to UVR where larger individuals tended to stay deeper in the water column, whereas smaller individuals showed strong preferences for being closer to the surface. Alteration in behavioral traits, such as vertical migration, allow zooplankton to escape from the threats instantly after exposure, whereas morphological defenses, for example, a smaller size, require longer time to establish. Large zooplankton prey species are more vulnerable to fish predators (Brooks and Dodson 1965) and thereby exhibit strong behavioral response, which is also supported in a study by Hansson and Hylander (2009*b*) who saw a deeper distribution of larger daphniids in the presence of predation risk. As smaller individuals are less vulnerable to predation, a weak, or even absent, vertical migration can save the cost of leaving the warm and food‐rich waters. Therefore, small animals such as invertebrate zooplankton are able to make risk assessments based on their size and respond accordingly.

In summary, we show that *D. magna* adopt divergent strategies over generations to handle multiple threats from predation and UVR, such as reducing their size to counteract predation risk and changing their behavior to avoid UVR damage. UVR‐exposed individuals showed a less pronounced behavioral response when re‐exposed to UVR, but this was associated with a cost of reduced clutch size during the first two generations. However, we show here, for the first time, that *D. magna* gradually restore the reproductive output and are able to reproduce and behave in a similar way as siblings reared under less stressful conditions already after three generations. We argue that transgenerational plasticity may be the underlying mechanism responsible for our results, which enable these invertebrates to meet changing environmental conditions rapidly, and thereby counteract extinction both at the local and the global scale.

## Supporting information

Appendix S1
