# Supplementary material for: Diverging responses to threats across generations in zooplankton
Source: Ecology. 2020 Aug 19;101(11):e03145. doi: 10.1002/ecy.3145 (PMC7685145; doi:10.1002/ecy.3145)
Supplement: Supplementary file 1 — Appendix S1 [file ECY-101-e03145-s001.pdf]

Supporting Information. Sha, Y., S. Tesson, and L.-A. Hansson 2020. Diverging responses to threats across generations in zooplankton. Ecology.

Appendix S1

Figure S1. Overview of the experimental design. *Daphnia magna* individuals from the three clones were exposed to control (C: No UVR or fish cue), predation (P: No UVR but with fish cue) and UVR (U: UVR without fish cue), in isolation and in combination (PU: UVR with fish cue) for three generations (G1 to G3). Each generation of the three clones was exposed to all treatments.

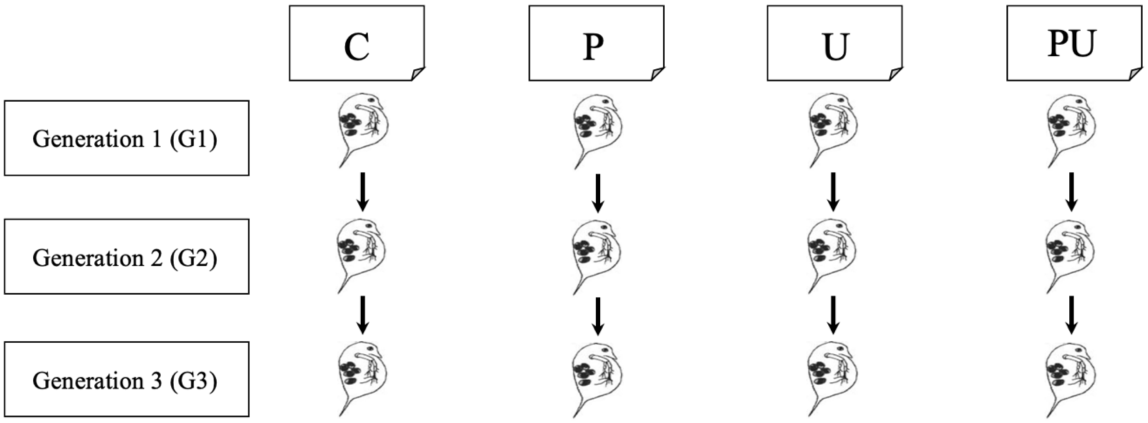

Figure S2. Ephippia formation. Percentage of *D. magna* females with ephippia in the different treatments (control, predation, UVR and the combination of predation and UVR) from generation one to three (G1 to G3).

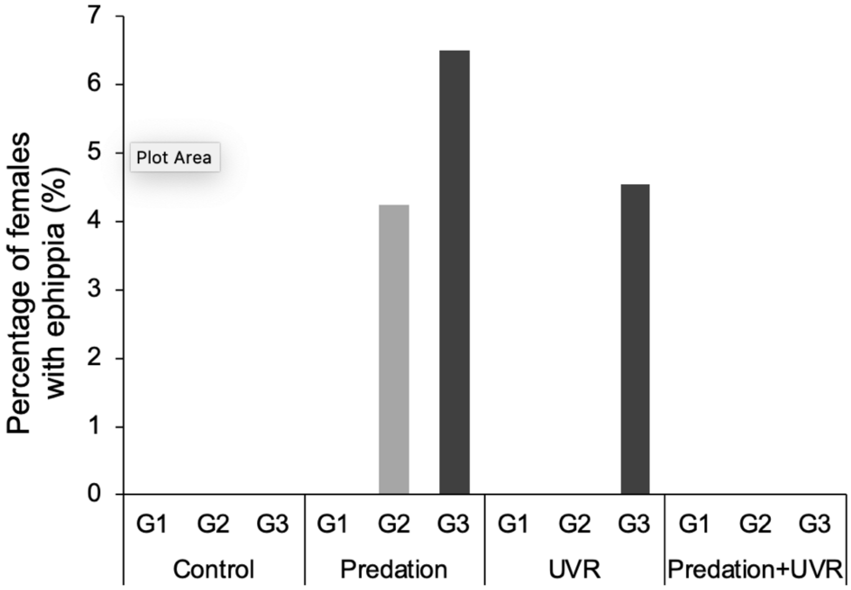

Figure S3. Female age and clutch size. Spearman correlations between the clutch size (i.e. total number of offspring produced from the first two clutches) and age of *D. magna* when clutch #2 was born for three generations (G1-G3). The results showed no significant relationship between the two variables, G1: Spearman $r = 0.192$, $p = 0.077$; G2: Spearman $r = 0.063$, $p = 0.399$; G3: Spearman $r = 0.026$, $p = 0.666$.

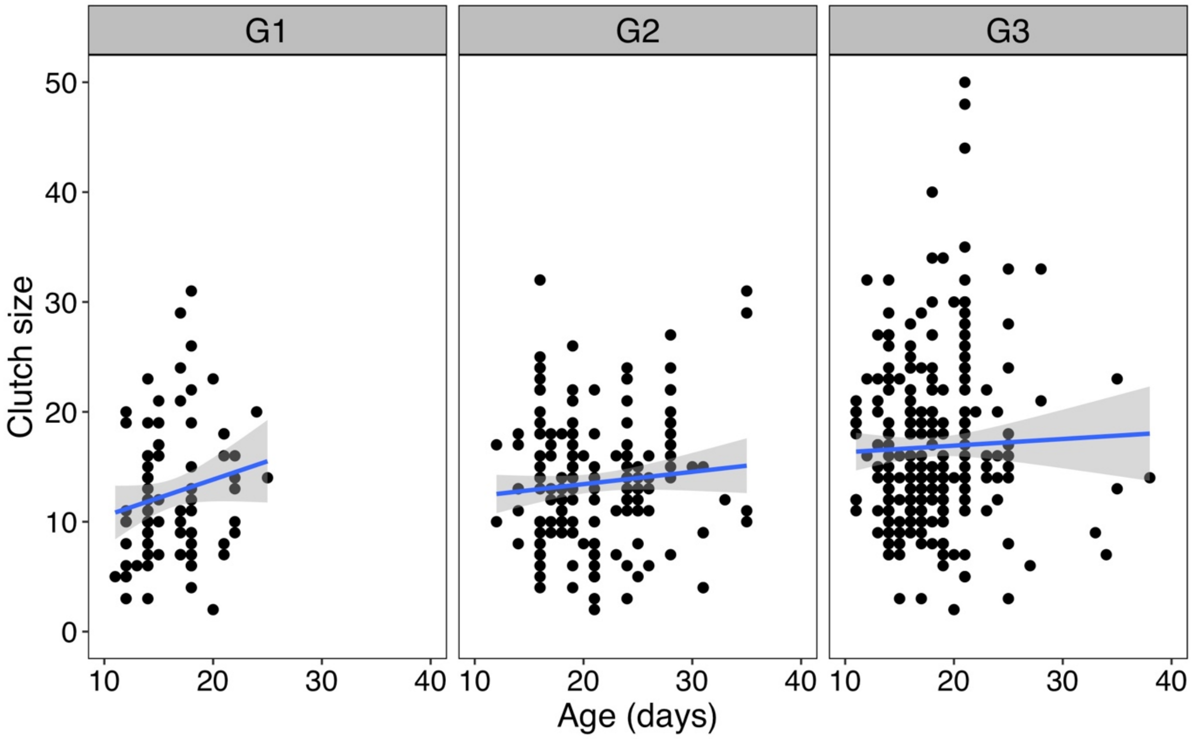

Table S1. Summary of morphology (body length), behavior (refuge demand and swimming speed) and reproduction (clutch size) over three generations for each treatment. Values are means (±1SD). Number of replicates are given in parentheses. Clutch size is given as the total number of offspring from the first two clutches.

Treatment	Generation	Body length (mm)	Refuge demand	Swimming speed (mm/s)	Clutch size
Control	G1	3.27 ± 0.06 (8)	28571.18 ± 6104.14 (7)	21.46 ± 2.36 (7)	11.45 ± 4.30 (22)
Control	G2	3.24 ± 0.12 (21)	29318.69 ± 7455.34 (21)	20.95 ± 4.66 (21)	12.44 ± 4.77 (39)
Control	G3	3.16 ± 0.14 (40)	25868.09 ± 5021.43 (38)	21.16 ± 4.12 (38)	14.29 ± 5.49 (65)
Predation	G1	3.32 ± 0.07 (9)	24932.12 ± 8060.00 (9)	19.10 ± 2.91 (9)	17.10 ± 5.42 (20)
Predation	G2	3.04 ± 0.20 (21)	22656.81 ± 6742.32 (20)	20.17 ± 6.14 (20)	13.89 ± 4.76 (47)
Predation	G3	2.95 ± 0.22 (27)	18606.58 ± 9416.24 (25)	20.95 ± 5.30 (25)	17.06 ± 6.80 (77)
UVR	G1	3.30 ± 0.09 (8)	15778.64 ± 5651.68 (6)	15.30 ± 1.27 (6)	8.56 ± 4.07 (16)
UVR	G2	3.23 ± 0.11 (21)	14827.03 ± 10041.89 (17)	16.99 ± 3.78 (17)	11.23 ± 5.27 (39)
UVR	G3	3.29 ± 0.17 (38)	12634.05 ± 7240.01 (24)	18.06 ± 6.71 (24)	16.03 ± 8.18 (66)
Predation+UVR	G1	3.30 ± 0.11 (8)	18752.19 ± 12129.91 (8)	17.04 ± 3.19 (8)	12.50 ± 6.55 (28)
Predation+UVR	G2	3.24 ± 0.12 (16)	18132.33 ± 10433.77 (16)	18.64 ± 4.92 (16)	15.70 ± 6.44 (54)
Predation+UVR	G3	3.19 ± 0.19 (28)	14213.44 ± 10086.86 (25)	19.01 ± 4.45 (25)	19.24 ± 8.14 (80)

Table S2. Further pairwise comparisons, body length of *Daphnia magna*. Tukey's post hoc tests were run following the linear mixed effect models to compare 1) changes in body length over three generations for each treatment and 2) differences in body length among treatments for each generation. Bold font indicates significant results ($p < 0.05$).

Contrast	Estimate	t-ratio	*p*
Changes in body length over three generations for each treatment			
Treatment = Control			
G1 - G2	0.026	0.406	0.913
G1 - G3	0.099	1.659	0.223
G2 - G3	0.073	1.759	0.186
Treatment = Predation			
G1 - G2	0.266	4.314	**<.0001**
G1 - G3	0.369	6.192	**<.0001**
G2 - G3	0.104	2.292	0.059
Treatment = UVR			
G1 - G2	0.073	1.132	0.496
G1 - G3	0.019	0.320	0.945
G2 - G3	-0.053	-1.270	0.414
Treatment = Predation+UVR			
G1 - G2	0.057	0.851	0.672
G1 - G3	0.106	1.710	0.204
G2 - G3	0.049	1.008	0.573
Differences in body length among treatments for each generation			
Generation 1			
Control - Predation	-0.040	-0.533	0.951
Control - UVR	-0.025	-0.320	0.989
Control - Predation+UVR	-0.026	-0.339	0.987
Predation - UVR	0.015	0.204	0.997
Predation - Predation+UVR	0.014	0.184	0.998
UVR - Predation+UVR	-0.001	-0.019	1.000
Generation 2			
Control - Predation	-0.040	-0.533	0.951
Control - UVR	-0.025	-0.320	0.989
Control - Predation+UVR	-0.026	-0.339	0.987
Predation - UVR	0.015	0.204	0.997
Predation - Predation+UVR	0.014	0.184	0.998
UVR - Predation+UVR	-0.001	-0.019	1.000
Generation 3			
Control - Predation	0.230	5.931	**<.0001**
Control - UVR	-0.105	-2.958	**0.018**
Control - Predation+UVR	-0.020	-0.514	0.956
Predation - UVR	-0.335	-8.586	**<.0001**
Predation - Predation+UVR	-0.250	-5.955	**<.0001**
UVR - Predation+UVR	0.085	2.197	0.127

Table S3. Further pairwise comparisons, refuge demand of *Daphnia magna*. Tukey's post hoc tests were run following the linear mixed effect models to compare 1) changes in refuge demand over three generations for each treatment and 2) differences in refuge demand among treatments for each generation. Bold font indicates significant results ($p < 0.05$).

Contrast	Estimate	t-ratio	*p*
Changes in refuge demand over three generations for each treatment			
Treatment = Control			
G1 - G2	-0.024	-0.071	0.997
G1 - G3	0.070	0.217	0.974
G2 - G3	0.094	0.441	0.898
Treatment = Predation			
G1 - G2	0.126	0.402	0.915
G1 - G3	0.353	1.160	0.479
G2 - G3	0.227	0.961	0.602
Treatment = UVR			
G1 - G2	0.558	1.509	0.289
G1 - G3	0.519	1.458	0.313
G2 - G3	-0.039	-0.158	0.986
Treatment = Predation+UVR			
G1 - G2	-0.045	-0.134	0.990
G1 - G3	0.354	1.119	0.504
G2 - G3	0.400	1.593	0.251
Differences in refuge demand among treatments for each generation			
Generation 1			
Control - Predation	0.121	0.306	0.990
Control - UVR	0.469	1.073	0.707
Control - Predation+UVR	0.678	1.681	0.336
Predation - UVR	0.348	0.844	0.833
Predation - Predation+UVR	0.558	1.473	0.456
UVR - Predation+UVR	0.210	0.495	0.960
Generation 2			
Control - Predation	0.271	1.111	0.683
Control - UVR	1.051	4.092	**<.0001**
Control - Predation+UVR	0.657	2.534	0.058
Predation - UVR	0.781	3.005	**0.016**
Predation - Predation+UVR	0.386	1.476	0.454
UVR - Predation+UVR	-0.394	-1.434	0.480
Generation 3			
Control - Predation	0.404	1.999	0.192
Control - UVR	0.918	4.396	**<.0001**
Control - Predation+UVR	0.963	4.799	**<.0001**
Predation - UVR	0.515	2.288	0.104
Predation - Predation+UVR	0.559	2.523	0.060
UVR - Predation+UVR	0.045	0.195	0.997

Table S4. Further pairwise comparisons, swimming speed of *Daphnia magna*. Tukey's post hoc tests were run following the linear mixed effect models to compare 1) changes in speed over three generations for each treatment and 2) differences in speed among treatments for each generation. Bold font indicates significant results ($p < 0.05$).

Contrast	Estimate	t-ratio	*p*
Changes in speed over three generations for each treatment			
Treatment = Control			
G1 - G2	0.480	0.229	0.972
G1 - G3	0.280	0.142	0.989
G2 - G3	-0.200	-0.153	0.987
Treatment = Predation			
G1 - G2	-0.989	-0.513	0.865
G1 - G3	-1.862	-0.996	0.580
G2 - G3	-0.873	-0.603	0.819
Treatment = UVR			
G1 - G2	-1.665	-0.730	0.746
G1 - G3	-2.802	-1.277	0.410
G2 - G3	-1.138	-0.747	0.736
Treatment = Predation+UVR			
G1 - G2	-1.587	-0.763	0.726
G1 - G3	-1.923	-0.985	0.587
G2 - G3	-0.336	-0.218	0.974
Differences in speed among treatments for each generation			
Generation 1			
Control - Predation	2.251	0.930	0.789
Control - UVR	5.934	2.213	0.123
Control - Predation+UVR	4.355	1.752	0.300
Predation - UVR	3.683	1.452	0.468
Predation - Predation+UVR	2.104	0.902	0.804
UVR - Predation+UVR	-1.580	-0.608	0.930
Generation 2			
Control - Predation	0.782	0.521	0.954
Control - UVR	3.790	2.407	0.079
Control - Predation+UVR	2.288	1.434	0.480
Predation - UVR	3.008	1.890	0.236
Predation - Predation+UVR	1.506	0.934	0.786
UVR - Predation+UVR	-1.502	-0.892	0.809
Generation 3			
Control - Predation	0.109	0.088	1.000
Control - UVR	2.852	2.247	0.114
Control - Predation+UVR	2.152	1.741	0.306
Predation - UVR	2.743	1.988	0.196
Predation - Predation+UVR	2.043	1.500	0.440
UVR - Predation+UVR	-0.700	-0.504	0.958

Table S5. Further pairwise comparisons, clutch size of *Daphnia magna*. Tukey's post hoc tests were run following the linear mixed effect models to compare 1) changes in clutch size over three generations for each treatment and 2) differences in clutch size among treatments for each generation. Bold font indicates significant results ($p < 0.05$).

Contrast	Estimate	t-ratio	*p*
**Changes in clutch size over three generations for each treatment**			
Treatment = Control			
G1 - G2	-0.120	-0.560	0.842
G1 - G3	-0.341	-1.719	0.199
G2 - G3	-0.221	-1.352	0.367
Treatment = Predation			
G1 - G2	0.460	2.145	0.082
G1 - G3	0.041	0.206	0.977
G2 - G3	-0.418	-2.814	**0.014**
Treatment = UVR			
G1 - G2	-0.413	-1.734	0.194
G1 - G3	-1.061	-4.741	**<.0001**
G2 - G3	-0.648	-3.995	**<.0001**
Treatment = Predation+UVR			
G1 - G2	-0.421	-2.246	0.065
G1 - G3	-0.873	-4.949	**<.0001**
G2 - G3	-0.451	-3.185	**0.004**
**Differences in clutch size among treatments for each generation**			
Generation 1			
Control - Predation	-0.730	-2.933	**0.018**
Control - UVR	0.494	1.870	0.242
Control - Predation+UVR	-0.080	-0.349	0.985
Predation - UVR	1.224	4.549	**<.0001**
Predation - Predation+UVR	0.650	2.761	**0.030**
UVR - Predation+UVR	-0.574	-2.279	0.104
Generation 2			
Control - Predation	-0.150	-0.861	0.825
Control - UVR	0.201	1.108	0.685
Control - Predation+UVR	-0.381	-2.258	0.109
Predation - UVR	0.351	2.015	0.184
Predation - Predation+UVR	-0.231	-1.444	0.473
UVR - Predation+UVR	-0.582	-3.449	**0.003**
Generation 3			
Control - Predation	-0.348	-2.567	0.051
Control - UVR	-0.226	-1.606	0.376
Control - Predation+UVR	-0.612	-4.549	**<.0001**
Predation - UVR	0.122	0.901	0.804
Predation - Predation+UVR	-0.264	-2.060	0.168
UVR - Predation+UVR	-0.386	-2.889	**0.021**
